# Supplementary material for: Urine based near-infrared spectroscopy analysis reveals a noninvasive and convenient diagnosis method for cancers: a pilot study
Source: PeerJ. 2023 Aug 31;11:e15895. doi: 10.7717/peerj.15895 (PMC10475272; doi:10.7717/peerj.15895)
Supplement: Supplemental Information 1 [file peerj-11-15895-s001.docx]

**R Codes for modeling**

#Packages needed

library(data.table)

library(dplyr)

library(caret)

library(hyperSpec)

library(Hmisc)

library(squash)

library(e1071)

library(signal)

library(pls)

#Function needed

toDF <- function(x){

l <- list()

for(i in 1:nrow(x)){

l[[i]] <- x[i,]

}

ldf <- rbindlist(l)

}

#Load data

load("./datawithPheno327.RData") # data loaded

t <- data # data contains all the NIRS information

t218 <- sample(327,218) # Randomly dividing a dataset into train and test sets

train <- data[t,] # train set without "scaling"

test <- data[-t,] # test set without "scaling"

#NIRS scaled with "center" and "scale"

prePro <- preProcess(train[,-c(1:6)],

method = c("center", "scale"))

trainM <- predict(prePro,train[,-c(1:6)]) #train set with "scaling"

testM <- predict(prePro,test[,-c(1:6)]) #test set with "scaling"

########################Preprocessing the NIRS raw data#######################

#Column1-6: labels; 7-1563: wavenumbers

wavenumber <- as.numeric(substr(names(train)[-c(1:6)],2,8))

mydata <- data.frame(data[,c(1:6)], NIR = I(data[,-c(1:6)]))

#Apply Savitzky-Golay smoothing to all spectra

newspectra<-apply(mydata$NIR,1, FUN=sgolayfilt, p = 2, n = 3, m = 0, ts = 1)

#Create new data frame:

mydataSmoothSG <- data.frame(mydata[,1:6], NIR = I(as.data.frame(t(newspectra)))) names(mydataSmoothSG$NIR) <- names(data[,-c(1:6)])

SG <- toDF(mydataSmoothSG$NIR)

#Baseline correction

#Convert mydata to an hyperSpec S4 object:

mydataHS <- new("hyperSpec", spc = as.matrix(mydata$NIR), wavelength = wavelengths)

#Compute baselines using order 2 polynomials:

baseline <- spc.fit.poly.below(fit.to = mydataHS, poly.order = 2)

mybaseline <- data.frame(mydata[,c(1:6)], NIR = I(baseline@data$spc))

newspectra <- mydataHS@data$spc-baseline@data$spc

mydataBSL<-data.frame(mydata[,1:6], NIR = I(newspectra))

l <- list()

for(i in 1:nrow(data)){

l[[i]] <- mydataBSL$NIR[i,]}

BSL <- as.data.frame(do.call(rbind,l))

names(BSL) <- names(data[,-c(1:6)])

#The first derivative

newspectra<-apply(mydata$NIR, 1, FUN=sgolayfilt, p = 2, n = 5, m = 1, ts = 1)

mydataDERIV1<-data.frame(mydata[,1:6], NIR = I(as.data.frame(t(newspectra))))

DERIV1 <- toDF(mydataDERIV1$NIR)

names(DERIV1) <- names(data[,-c(1:6)])

#The second derivative

newspectra<-apply(mydata$NIR, 1, FUN=sgolayfilt, p = 2, n = 5, m = 2, ts = 1)

mydataDERIV2<-data.frame(mydata[,1:6], NIR = I(as.data.frame(t(newspectra))))

DERIV2 <- toDF(mydataDERIV2$NIR)

names(DERIV2) <- names(data[,-c(1:6)])

#MSC correction

newspectra<-msc(as.matrix(mydata$NIR))

mydataMSC<-data.frame(mydata[,1:6], NIR = I(as.data.frame(newspectra)))

MSC <- toDF(mydataMSC$NIR)

names(MSC) <- names(data[,-c(1:6)])

###########################Modeling########################################

#train <- data[t,] # train set without "scaling"

#test <- data[-t,] # test set without "scaling"

#NIRS scaled with "center" and "scale"

#prePro <- preProcess(train[,-c(1:6)],

method = c("center", "scale"))

#trainM <- predict(prePro,train[,-c(1:6)]) #train set with "scaling"

#testM <- predict(prePro,test[,-c(1:6)]) #test set with "scaling"

trainclass <- as.factor(train$Class3)

testclass <- as.factor(test$Class3)

#Modeling with the NIRS different preprocessing methods, including scaling, SG, MSC, BSL, #DERIV1, DERIV2

#Modeling with raw data with scaling or without scaling

fitControl <- trainControl(# 5-fold CV, 10 repeats

method = "repeatedcv",

number = 5,

repeats = 10)

set.seed(101)

fit.pls.raw <- train(y = trainclass,

x = trainM,

# train[,-c(1:6)] used for modeling without scaling

method = "pls",

trControl = fitControl,

verbose = FALSE )

confusionMatrix(predict(fit.pls.raw,testM),testclass)

# test[,-c(1:6)] used for modeling without scaling

set.seed(101)

fit.svm.raw <- svm( y = trainclass,

x = trainM,

# train[,-c(1:6)] used for modeling without scaling

cross = 5,

kernel = "linear",

type = "C-classification",

scale =F,

probability= T,

cost = 10)# from 0.001 to 100）

confusionMatrix(predict(fit.svm.raw,testM),testclass)

# test[,-c(1:6)] used for modeling without scaling

#Modeling with NIRS with SG preprocessing

set <- SG

trainM <- set[t218,]

testM <- set[-t218,]

pre <- preProcess(trainM,method =c("center","scale"))

trainM <- predict(pre,trainM)

testM <- predict(pre,testM)

#pls

fitControl <- trainControl( # 5-fold CV, 10 repeats

method = "repeatedcv",

number = 5,

repeats = 10)

set.seed(101)

fit.pls.SG <- train(y = trainclass,

x = trainM,

method = "pls",

trControl = fitControl,

verbose = FALSE )

confusionMatrix(predict(fit.pls.SG,testM),testclass)

set.seed(101)

fit.svm.SG <- svm( y = trainclass,

x = trainM,

cross = 5,

kernel = "linear",

type = "C-classification",

scale =F,

probability= T,

cost = 10)# from 0.001 to 100）

confusionMatrix(predict(fit.svm.SG,testM),testclass)

#Modeling with raw data with MSC preprocessing

set <- MSC

trainM <- set[t218,]

testM <- set[-t218,]

# skip the following step when modeling without "scaling"

pre <- preProcess(trainM,method =c("center","scale"))

trainM <- predict(pre,trainM)

testM <- predict(pre,testM)

#pls

fitControl <- trainControl( ## 5-fold CV, 10 repeats

method = "repeatedcv",

number = 5,

repeats = 10)

set.seed(101)

fit.pls.MSC <- train(y = trainclass,

x = trainM,

method = "pls",

trControl = fitControl,

verbose = FALSE )

confusionMatrix(predict(fit.pls.MSC,testM),testclass)

set.seed(101)

fit.svm.MSC <- svm( y = trainclass,

x = trainM,

cross = 5,

kernel = "linear",

type = "C-classification",

scale =F,

probability= T,

cost = 10)# from 0.001 to 100）

confusionMatrix(predict(fit.svm.MSC,testM),testclass)

#Modeling with raw data with BSL preprocessing

set <- BSL

trainM <- set[t218,]

testM <- set[-t218,]

# skip the following step when modeling without "scaling"

pre <- preProcess(trainM,method =c("center","scale"))

trainM <- predict(pre,trainM)

testM <- predict(pre,testM)

#pls

fitControl <- trainControl( # 5-fold CV, 10 repeats

method = "repeatedcv",

number = 5,

repeats = 10)

set.seed(101)

fit.pls.BSL <- train(y = trainclass,

x = trainM,

method = "pls",

trControl = fitControl,

verbose = FALSE )

confusionMatrix(predict(fit.pls.BSL,testM),testclass)

set.seed(101)

fit.svm.BSL <- svm( y = trainclass,

x = trainM,

cross = 5,

kernel = "linear",

type = "C-classification",

scale =F,

probability= T,

cost = 10)# from 0.001 to 100）

confusionMatrix(predict(fit.svm.BSL,testM),testclass)

#Modeling with raw data with DERIV1 preprocessing

set <- DERIV1

trainM <- set[t218,]

testM <- set[-t218,]

# skip the following step when modeling without "scaling"

pre <- preProcess(trainM,method =c("center","scale"))

trainM <- predict(pre,trainM)

testM <- predict(pre,testM)

#pls

fitControl <- trainControl( # 5-fold CV, 10 repeats

method = "repeatedcv",

number = 5,

repeats = 10)

set.seed(101)

fit.pls.DERIV1 <- train(y = trainclass,

x = trainM,

method = "pls",

trControl = fitControl,

verbose = FALSE )

confusionMatrix(predict(fit.pls.DERIV1,testM),testclass)

set.seed(101)

fit.svm.DERIV1 <- svm( y = trainclass,

x = trainM,

cross = 5,

kernel = "linear",

type = "C-classification",

scale =F,

probability= T,

cost = 10)# from 0.001 to 100）

confusionMatrix(predict(fit.svm.DERIV1,testM),testclass)

#Modeling with raw data with DERIV2 preprocessing

set <- DERIV2

trainM <- set[t218,]

testM <- set[-t218,]

# skip the following step when modeling without "scaling"

pre <- preProcess(trainM,method =c("center","scale"))

trainM <- predict(pre,trainM)

testM <- predict(pre,testM)

#pls

fitControl <- trainControl( #5-fold CV, 10 repeats

method = "repeatedcv",

number = 5,

repeats = 10)

set.seed(101)

fit.pls.DERIV2 <- train(y = trainclass,

x = trainM,

method = "pls",

trControl = fitControl,

verbose = FALSE )

confusionMatrix(predict(fit.pls.DERIV2,testM),testclass)

set.seed(101)

fit.svm.DERIV2 <- svm( y = trainclass,

x = trainM,

cross = 5,

kernel = "linear",

type = "C-classification",

scale =F,

probability= T,

cost = 5)# from 0.001 to 100）

confusionMatrix(predict(fit.svm.DERIV2,testM),testclass)
