# Supplementary material for: Urine based near-infrared spectroscopy analysis reveals a noninvasive and convenient diagnosis method for cancers: a pilot study
Source: PeerJ. 2023 Aug 31;11:e15895. doi: 10.7717/peerj.15895 (PMC10475272; doi:10.7717/peerj.15895)
Supplement: Supplemental Information 3 [file peerj-11-15895-s003.docx]

**Table 1S: The cancer types in train set and test set.**

| **Train set** | |  | **Test set** | |
| --- | --- | --- | --- | --- |
| **Class** | **Number of cases** |  | **Class** | **Number of cases** |
| Bladder cancer | 2 |  | Breast cancer | 5 |
| Breast cancer | 5 |  | Cervical cancer | 2 |
| Cervical cancer | 17 |  | Colon cancer | 5 |
| Colon cancer | 9 |  | Gastric cancer | 17 |
| Gastric cancer | 15 |  | Kidney cancer | 1 |
| liver cancer | 4 |  | liver cancer | 5 |
| Lung cancer | 43 |  | Lung cancer | 19 |
| Nasopharyngeal carcinoma | 5 |  | Nasopharyngeal carcinoma | 4 |
| Ovarian cancer | 7 |  | Ovarian cancer | 4 |
| Thyroid cancer | 9 |  | Thyroid cancer | 3 |
| Healthy control | 102 |  | Healthy control | 44 |
