# Supplementary material for: Urine based near-infrared spectroscopy analysis reveals a noninvasive and convenient diagnosis method for cancers: a pilot study
Source: PeerJ. 2023 Aug 31;11:e15895. doi: 10.7717/peerj.15895 (PMC10475272; doi:10.7717/peerj.15895)
Supplement: Supplemental Information 5 [file peerj-11-15895-s005.docx]

**Table S3:** **Prediction performance of modeling with different pre-treatment methods (Training).**

| **Pretreatment ^a^** | **Model** | **Sensitivity** | **Specificity** | **PPV ^b^** | **NPV ^c^** | **Precision** | **Recall** | **Accuracy** |
| --- | --- | --- | --- | --- | --- | --- | --- | --- |
| Raw | PLS | 0.759 | 0.598 | 0.682 | 0.685 | 0.682 | 0.759 | 0.683 |
| SG | PLS | 0.759 | 0.598 | 0.682 | 0.685 | 0.682 | 0.759 | 0.683 |
| MSC | PLS | 0.716 | 0.559 | 0.648 | 0.633 | 0.648 | 0.716 | 0.642 |
| BSL | PLS | 0.750 | 0.529 | 0.644 | 0.651 | 0.644 | 0.750 | 0.647 |
| DERIV1 | PLS | 0.750 | 0.588 | 0.674 | 0.674 | 0.674 | 0.750 | 0.674 |
| DERIV2 | PLS | 0.862 | 0.676 | 0.752 | 0.812 | 0.752 | 0.862 | 0.775 |
| Raw | SVM | 0.741 | 0.627 | 0.694 | 0.681 | 0.694 | 0.741 | 0.688 |
| SG | SVM | 0.741 | 0.627 | 0.694 | 0.681 | 0.694 | 0.741 | 0.688 |
| MSC | SVM | 0.776 | 0.598 | 0.687 | 0.701 | 0.687 | 0.776 | 0.693 |
| BSL | SVM | 0.810 | 0.569 | 0.681 | 0.725 | 0.681 | 0.810 | 0.697 |
| DERIV1 | SVM | 0.888 | 0.324 | 0.599 | 0.717 | 0.599 | 0.888 | 0.624 |
| DERIV2 | SVM | 0.905 | 0.578 | 0.709 | 0.843 | 0.709 | 0.905 | 0.752 |
| Raw+scaled | PLS | 0.784 | 0.598 | 0.689 | 0.709 | 0.689 | 0.784 | 0.697 |
| SG+scaled | PLS | 0.784 | 0.598 | 0.689 | 0.709 | 0.689 | 0.784 | 0.697 |
| MSC+scaled | PLS | 0.707 | 0.676 | 0.713 | 0.670 | 0.713 | 0.707 | 0.693 |
| BSL+scaled | PLS | 0.750 | 0.569 | 0.664 | 0.667 | 0.664 | 0.750 | 0.665 |
| DERIV1+scaled | PLS | 0.879 | 0.667 | 0.750 | 0.829 | 0.750 | 0.879 | 0.780 |
| DERIV2+scaled | PLS | 0.983 | 0.941 | 0.950 | 0.980 | 0.950 | 0.983 | 0.963 |
| Raw+scaled | SVM | 0.845 | 0.863 | 0.875 | 0.830 | 0.875 | 0.845 | 0.853 |
| SG+scaled | SVM | 0.845 | 0.863 | 0.875 | 0.830 | 0.875 | 0.845 | 0.853 |
| MSC+scaled | SVM | 0.957 | 0.961 | 0.965 | 0.951 | 0.965 | 0.957 | 0.959 |
| BSL+scaled | SVM | 1.000 | 0.980 | 0.983 | 1.000 | 0.983 | 1.000 | 0.991 |
| DERIV1+scaled | SVM | 1.000 | 1.000 | 1.000 | 1.000 | 1.000 | 1.000 | 1.000 |
| DERIV2+scaled | SVM | 1.000 | 1.000 | 1.000 | 1.000 | 1.000 | 1.000 | 1.000 |

^a^: Raw-with no pretreatment, SG- Savitsky-Golay smoothing, MSC- multiplicative scatter correction, BSL-baseline removal, DERIV1-the first derivative, DERIV2- the second derivative.

^b^: Positive prediction value (PPV) = (true positive)/(true positive + false positive).

c: Negative prediction value (NPV) = (true negative)/(true negative + false negative).
